# Cholestasis of Sepsis: A Case Report

**DOI:** 10.7759/cureus.8897

**Published:** 2020-06-29

**Authors:** Atit Ghoda, Manoj Ghoda

**Affiliations:** 1 General Internal Medicine, East Surrey Hospital/Surrey and Sussex Healthcare National Health Service Trust, Redhill, GBR; 2 Gastroenterology and Hepatology, Gujarat Superspeciality Clinic, Ahmedabad, IND

**Keywords:** tuberculosis, mediastinal abscess

## Abstract

A 46-year-old man presented with fever, general lethargy, and weight loss over the last few months. He started to develop jaundice and his condition worsened. Blood tests confirmed rising levels of conjugated bilirubin with near-normal alanine aminotransferase, alkaline phosphatase, and prothrombin time. Imaging of the liver and biliary system, including ultrasound, computed tomography (CT), and magnetic resonance cholangiopancreatography (MRCP), did not show any focal lesion or biliary obstruction. Human immunodeficiency virus (HIV) and hepatitis screening were negative. A chest x-ray showed no consolidation. An echocardiogram showed no evidence of endocarditis. An ultrasound of the neck and axilla did not show any enlarged lymph nodes. A chest CT scan revealed a mediastinal abscess that contained acid-fast bacilli when aspirated and stained. The patient was started on first-line antituberculous treatment. The jaundice was thought to be secondary to cholestasis of sepsis and resolved completely over the subsequent weeks. His bilirubin levels returned to normal after treatment initiation.

## Introduction

The presence of elevated conjugated bilirubin levels is not an unusual anomaly in patients presenting with various infections. Complex mechanisms exist in the liver for the production, transport, and excretion of bile. Sepsis and infections can lead to alterations in these pathways and lead to cholestasis and jaundice.

## Case presentation

A 46-year-old male presented to his general practitioner in India with complaints of easy fatigability, malaise, body aches, and reduced appetite over the last four months. He had no known medical history and did not take any regular medication. He had no history of smoking, alcohol consumption, or any recreational drug use. There was no noteworthy family history.

Upon review, he was noted to have a high body mass index (BMI) and a weight of 103 kg. There was no anemia, cyanosis, or jaundice. No lymph nodes were palpable. Systems review was unremarkable.

Initial investigations were as follows (reference range in parentheses): hemoglobin (Hb) 13 g/dL (14 - 17 g/dL), erythrocyte sedimentation rate (ESR) 31 mm/h (< 15 mm/h), bilirubin 16 µmol/L (5.1 - 20.5 µmol/L), alanine aminotransferase (ALT) 38 units/L (0 - 35 units/L), serum aspartate aminotransferase (AST) 35 units/L (0 - 35 units/L), and alkaline phosphatase (ALP) 118 units/L (36 - 92 units/L). Electrolytes and renal function were normal. Chest x-ray and electrocardiogram (ECG) were reported to be normal.

His condition did not improve over the following three weeks and his weight dropped from 103 kg to about 95 kg. Repeat blood tests were unremarkable. Transthoracic echocardiography did not show any evidence of endocarditis. Glucose tolerance tests, thyroid function tests, and human immunodeficiency virus (HIV) and viral hepatitis screening tests were negative.

He remained unwell over the next couple of weeks. He began feeling feverish at night with a further bodyweight loss of 3 kg and subsequently developed jaundice. Repeat blood test results included a Hb of 11.2 g/dL (14 - 17 g/dL), mean corpuscular volume (MCV) 82 fL (80 - 100 fL), white cell count (WCC) 5.6 x 10^9^/L (4.0 - 10 x 10^9^/L), ESR 35 mm (0 - 15 mm/h), bilirubin 53 µmol/L (70% conjugated) (5.1 - 20.5 µmol/L), ALT 69 units/L (0 - 35 units/L), AST 72 units/L (0 - 35 units/L), ALP 136 units/L (36 - 92 units/L), and albumin 33 g/L (35 - 55 g/L). Prothrombin time (PT) was 14/13 sec (11 - 13 sec) with an international normalized ratio (INR) of 1.1 (0.9 - 1.2). Blood cultures did not show any growth. A tuberculin test showed an induration of 22 mm at 72 hours. Abdominal ultrasound revealed a normal spleen, pancreas, kidneys, liver, and gallbladder with no evidence of biliary dilatation. No lymph nodes were detected. Ultrasound of the neck and axilla for any lymph nodes was normal, and no abnormality was detected on repeat chest radiography.

His symptoms did not improve over the next few days and his condition progressively worsened. The patient felt frail, feverish (with a temperature of up to 39°C), and his jaundice had progressed. Bilirubin rose to 222 µmol/L (70% conjugated) and other relevant test results were ALT 76 units/L, AST 60 units/L, ALP 132 units/L, PT 15/13 sec with an INR of 1.2, and serum albumin 31 g/L (Table 1).

**Table 1:** Investigations and Approximate Timeline ALT: alanine aminotransferase; AST: serum aspartate aminotransferase; ESR: erythrocyte sedimentation rate; HIV: human immunodeficiency virus; INR: international normalized ratio; K: potassium; MCV: mean corpuscular volume; Na: sodium; T: time of first presentation to his general practitioner

Investigation	T = 0	T + 5 weeks	T + 7 weeks
Hb (g/dL)	13	11.2	
MCV (fL)	84	82	
ESR (mm/h)	31	35	
Na (mEq/l)	143	140	
K (mEq/l)	4.5	4.2	
Bilirubin ( µ mol/L)	16	53	222
ALT (units/L)	38	69	76
AST (units/L)	35	72	60
Alkaline phosphatase (units/L)	118	136	132
Albumin (g/L)		33	31
INR		1.1	1.2
Prothrombin time (PT) (sec)		14 (13)	15 (13)
HIV and viral hepatitis screening	Negative		
Thyroid function test	Normal		

Magnetic resonance cholangiopancreatography (MRCP), abdominal CT, and repeat abdominal ultrasound did not reveal any abnormality of the liver parenchyma or the biliary tree.

At this point, he was referred to our center for further assessment and management. On clinical examination, he appeared unwell and fatigued and seemed to have had a substantial weight loss. He was deeply jaundiced. No lymph nodes were palpable. There was no pedal edema or cyanosis. The abdomen was soft and scaphoid. The liver, spleen, and kidneys were not palpable. There were no ascites. Bowel sounds were present and regular. The rectal examination was unremarkable. The stool appeared yellow and was negative for occult blood.

Respiratory system examination revealed tachypnoea with a respiratory rate of 24 breaths/minute. His chest expanded symmetrically and air entry was good and equal with bilateral vesicular breathing sounds. The cardiovascular system was unremarkable with normal heart sounds.

The biochemical picture, including elevated bilirubin with near-normal liver enzyme levels, has been seen in cholestasis related to inflammation or sepsis. There was a temporal relation with symptoms that were suggestive of infection and inflammation. Efforts were, therefore, made to locate the source.

The length of the illness suggested a chronic disease. Progressive weight loss and low-grade fever suggested tuberculosis amongst a few other possible causes, such as brucellosis, but the focus was not found on routine examination.

**Figure 1:**
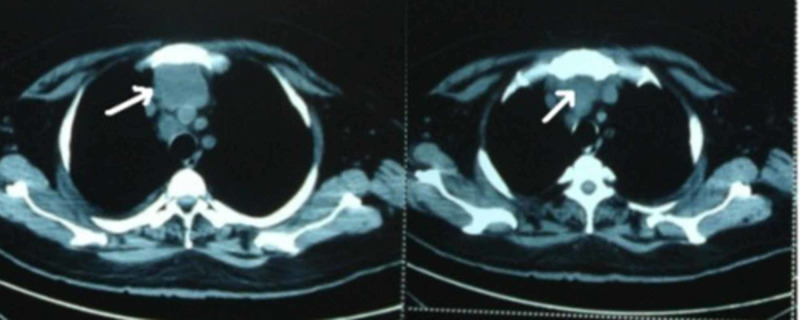
Mediastinal abscess before and after aspiration

In view of the tachypnea with no clear visible focal consolidation in the chest, chest high-resolution computed tomography (HRCT) was performed and revealed a large mediastinal abscess which, on aspiration, confirmed the presence of tuberculous bacteria (Figure 1).

The patient underwent needle aspiration of the tuberculous abscess and was started on isoniazid, rifampicin, pyrazinamide, and ethambutol with regular liver enzyme monitoring.

He progressively became afebrile and regained his appetite, which resulted in weight stabilization. His liver functions progressively improved, and by three months, his bilirubin returned to within normal range (18.81 µmol/L), along with ALT (27 units/L), AST (25 units/L), and ALP (112 units/L) levels.

**Figure 2:**
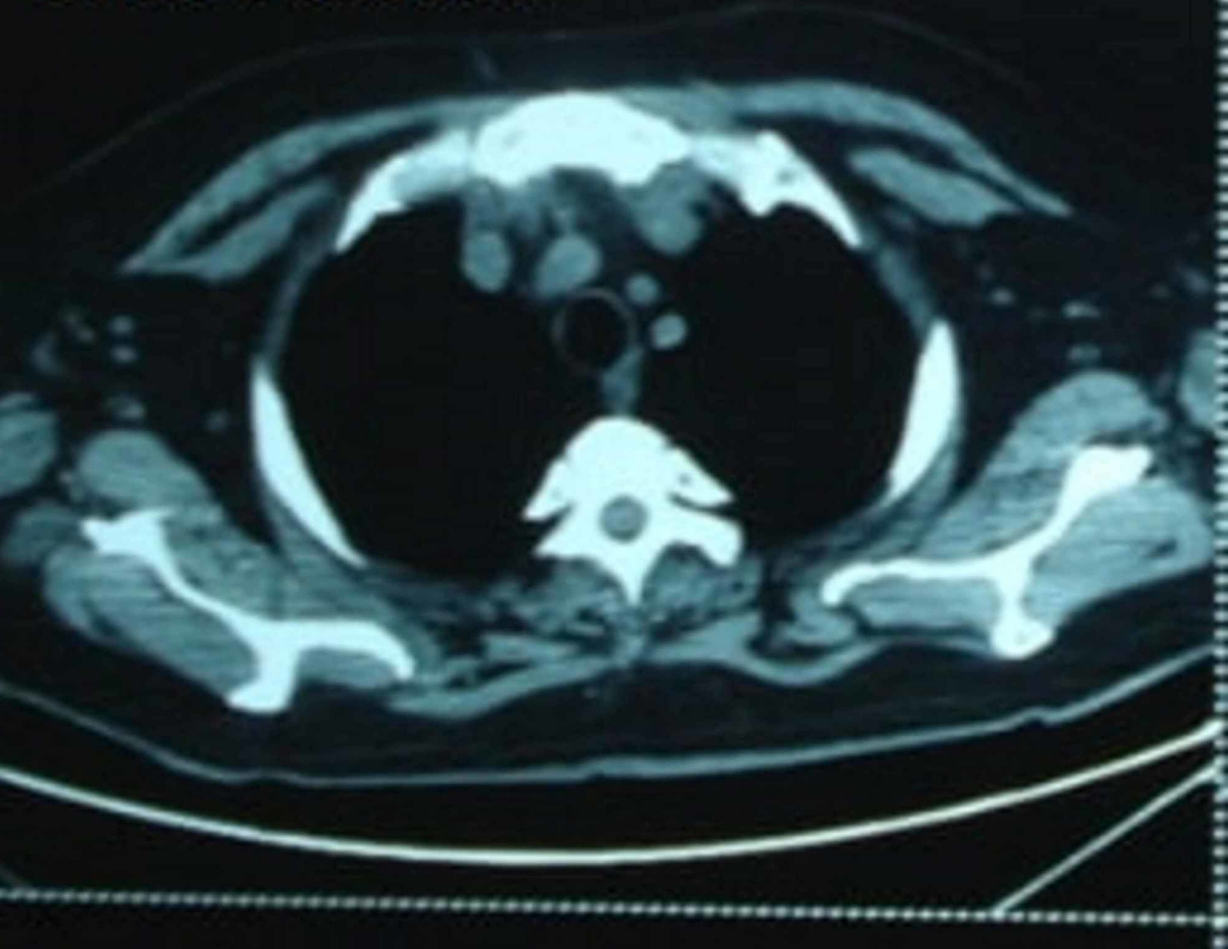
Computed tomography (CT) scan six months later showing resolution of the mediastinal abscess

By six months, his CT scan showed complete resolution of the mediastinal abscess (Figure 2).

## Discussion

Derangement of liver enzymes and hyperbilirubinemia in sepsis can be related to various mechanisms. Direct infections of the liver or biliary tree can lead to elevated bilirubin and deranged liver enzymes. Hypotension secondary to sepsis may lead to liver injury. Elevated bilirubin may also be observed during sepsis in response to circulating microbial products and is known as the cholestasis of sepsis [1].

The liver plays a key role in the metabolism of bilirubin. Complex cellular pathways are involved in the production and transport of bile acids. Cholestasis of sepsis appears to be related to altered homeostasis of these bile acids. It has been proposed that cytokines are potential drivers of these cholestatic alterations. It is not clear whether this phenomenon of cholestasis is harmful on its own or whether it provides any survival benefit [2-3].

The existence of such a condition has long been noted. In 1899, in his textbook, "The Principles and Practice of Medicine", Osler reported that pneumonia could lead to jaundice ("toxemic jaundice"): “In this form, there is no obstruction in the bile-passages, but the jaundice is associated with toxic states of the blood, dependent upon various poisons which either act directly on the blood itself or in some cases on the liver cells as well” [4].

Due to the lack of a precise clinical definition and heterogeneity of presentations, it is difficult to provide an accurate estimate of the actual incidence of this condition. It has been suggested, however, that sepsis is one of the most common causes of jaundice in ICU patients [5]. 

Laboratory abnormalities include the presence of hyperbilirubinemia with near-normal or mildly elevated aminotransferases and alkaline phosphatase. Imaging modalities, which include ultrasound, CT, and MRCP, are used to evaluate the liver and biliary tree to rule out any biliary abnormalities, focal lesions, and abscesses.

Cholestasis of sepsis does not require any specific treatment and jaundice is expected to improve as the sepsis resolves [6].

## Conclusions

Cholestasis of sepsis may occur in any form of extra-hepatic infection. Conjugated hyperbilirubinemia with normal or near-normal liver enzyme levels and alkaline phosphatase are the biochemical hallmark of this condition. Imaging of the liver and biliary tract can be used to find and rule out focal lesions and biliary obstruction. The cholestasis per se may not need any specific treatment. Jaundice is expected to improve as the sepsis resolves.
